# Experimental Models of Status Epilepticus and Neuronal Injury for Evaluation of Therapeutic Interventions

**DOI:** 10.3390/ijms140918284

**Published:** 2013-09-05

**Authors:** Doodipala Samba Reddy, Ramkumar Kuruba

**Affiliations:** Department of Neuroscience and Experimental Therapeutics, College of Medicine, Texas A&M University Health Science Center, 8447 State Highway 47, MREB Building, Bryan, TX 77807, USA

**Keywords:** perforant stimulation, pilocarpine, kainic acid, epilepsy, seizure, DFP, neurodegeneration

## Abstract

This article describes current experimental models of status epilepticus (SE) and neuronal injury for use in the screening of new therapeutic agents. Epilepsy is a common neurological disorder characterized by recurrent unprovoked seizures. SE is an emergency condition associated with continuous seizures lasting more than 30 min. It causes significant mortality and morbidity. SE can cause devastating damage to the brain leading to cognitive impairment and increased risk of epilepsy. Benzodiazepines are the first-line drugs for the treatment of SE, however, many people exhibit partial or complete resistance due to a breakdown of GABA inhibition. Therefore, new drugs with neuroprotective effects against the SE-induced neuronal injury and degeneration are desirable. Animal models are used to study the pathophysiology of SE and for the discovery of newer anticonvulsants. In SE paradigms, seizures are induced in rodents by chemical agents or by electrical stimulation of brain structures. Electrical stimulation includes perforant path and self-sustaining stimulation models. Pharmacological models include kainic acid, pilocarpine, flurothyl, organophosphates and other convulsants that induce SE in rodents. Neuronal injury occurs within the initial SE episode, and animals exhibit cognitive dysfunction and spontaneous seizures several weeks after this precipitating event. Current SE models have potential applications but have some limitations. In general, the experimental SE model should be analogous to the human seizure state and it should share very similar neuropathological mechanisms. The pilocarpine and diisopropylfluorophosphate models are associated with prolonged, diazepam-insensitive seizures and neurodegeneration and therefore represent paradigms of refractory SE. Novel mechanism-based or clinically relevant models are essential to identify new therapies for SE and neuroprotective interventions.

## 1. Introduction

### 1.1. Epilepsy

Epilepsy is a chronic condition characterized by recurrent unprovoked seizures. It affects about 3 million people in the United States and approximately 65 million people worldwide [1,2]. Epilepsy affects people of all ages and both genders. Every year, nearly 150,000 new cases of epilepsy are diagnosed in the United States [2]. A seizure is an abnormal electrical discharge in the brain that causes alteration in consciousness, sensation, and behavior. When the risk of spontaneous seizures is sufficiently high, generally after two spontaneous seizures, the patient is diagnosed with epilepsy. However, a normal EEG never rules out the diagnosis of epilepsy. Epilepsy is a disorder with many possible causes. Epilepsy may develop because of an abnormality in brain wiring, an imbalance in inhibitory and excitatory neurotransmitters, or some combination of these factors. Primary epilepsy (50%) is idiopathic (“unknown cause”). In secondary epilepsy (50%), referred as acquired epilepsy, seizures may result from a variety of conditions including trauma, anoxia, metabolic imbalances, tumors, encephalitis, drug withdrawal seizures, or neurotoxicity. The most common risk factors for epilepsy are cerebrovascular disease, brain tumors, alcohol, traumatic head injuries, malformations of cortical development, genetic inheritance, and infections of the central nervous system [1].

**Figure 1:**
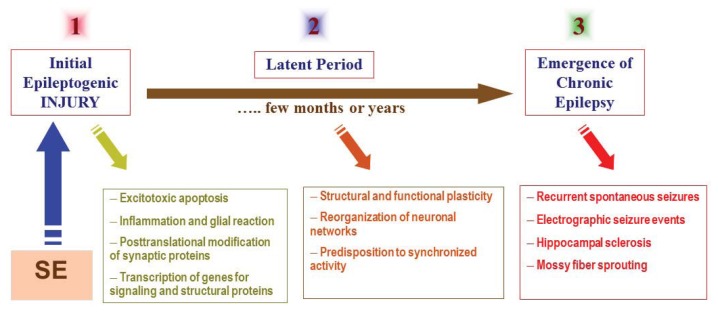
The pathophysiological basis of epileptogenesis following SE episode. The mechanisms involved in epileptogenesis involve an interaction of acute and delayed anatomic, molecular, and physiological events that are both complex and multifaceted. SE-induced neuronal injury activates diverse signaling events, such as inflammation, oxidation, apoptosis, neurogenesis and synaptic plasticity, which eventually leads to structural and functional changes in neurons. It is a progressive process that produces rearrangement of synaptic circuitry, neurogenesis, mossy fiber sprouting and a hyperexcitability state over weeks or months or years (latent period). These changes are eventually manifested as spontaneous recurrent seizures (epilepsy) in susceptible persons.

The mechanisms underlying development of epilepsy are not very well understood. The term “epileptogenesis” is used to describe the complex plastic changes in the brain that, following a precipitating insult or injury, convert a normal brain into a brain debilitated by recurrent seizures [3]. Current hypothesis about the epileptogenesis involves three stages (Figure 1): (i) the initial precipitating event; (ii) the latent period; and (iii) the chronic period with spontaneous seizures. Epileptogenesis is a slow process; it takes several months for spontaneous seizures to appear [4,5]. The time required for seizure occurrence (latent period) represents a window of opportunity for testing interventions in people at high risk for epilepsy [6,7]. Neuronal injury and neuroinflammation have been proposed to play a central role in the overall pathogenesis of acquired epilepsy (Figure 1).

Epilepsy is a spectrum disorder. It is the fourth most common neurological disorders in the US after migraine, stroke, and Alzheimer’s disease [2]. Epileptic seizures are classified into *partial* seizures (60% of all epilepsies), those beginning focally in a cortical site, and *generalized* seizures (40% of all epilepsies), those that involve both hemispheres widely from the outset [8]. Temporal lobe epilepsy (TLE) is the most common form of partial epilepsy, likely affecting at least 20% of all patients with epilepsy [8,9]. TLE is the most common form of drug-refractory epilepsy [10]. Atrophy of mesial temporal structures is well known to be associated with TLE and hippocampal sclerosis is the most frequent histological abnormality in this form of epilepsy [11]. Several antiepileptic drugs (AEDs) are available for the treatment of epilepsy (Table 1). AEDs act on diverse molecular targets to selectively modify the excitability of neurons by reducing the focal seizure discharges or preventing spread of excitation (Table 1). Despite many advances in epilepsy research, nearly 30% of people with epilepsy have “intractable seizures” that do not respond to even the best available AEDs.

### 1.2. Status Epilepticus

Status epilepticus (SE) is a life-threatening emergency characterized by a prolonged continuous state of convulsions. SE is defined as continuous seizure activity or multiple seizures without regaining consciousness for more than 30 min [12]. SE is a medical emergency in humans that if untreated, can lead to brain damage and death [13–15]. There are two types of SE, generalized convulsive SE and nonconvulsive SE. Untreated SE can result in death due to an inability of the brain to control and terminate the seizures. The pathophysiology of SE is not clearly understood but excess excitatory (glutamate) neurotransmission and loss of normal inhibitory (GABA) neurotransmission are thought to be the most likely mechanisms. The first-line therapies of choice are intravenous benzodiazepines (e.g., lorazepam and diazepam), which potentiate the inhibitory responses mediated by GABA-A receptors [16]. Intramuscular midazolam is equally effective as intravenous lorazepam for prehospital SE cessation [17]. However, the efficacy of benzodiazepines dramatically decreases with increasing durations of SE [18]. In some cases of SE, there is a complete loss of the therapeutic efficacy of benzodiazepines and more drastic second-line (phenytoin and fosphenytoin) and third-line therapies (propofol or phenobarbital) must be employed, but are not always successful [19]. These pharmacoresistant forms of SE are termed refractory SE. Refractory SE, which occurs in up to 40% of all patients with SE, remains a challenge for management because of poor prognosis [20–22]. In general, refractory SE is treated with coma induction using anesthetics such as propofol or pentobarbital [23]. Therefore, novel life-saving anticonvulsants are needed with improved profile for effective therapy of SE (Table 1).

Experimental models have been developed that mimic the continuous seizure state or SE. TLE can be induced with pharmacologic agents or by electrical stimulation in rodents. The common pharmacological models of SE are kainic acid [24,25], pilocarpine [26,27] and perforant path stimulation [28]. A single seizure episode is of short duration and usually self-limiting. During SE, seizure activity is not limited and seizures occur continuously with polyspiking detected by EEG. The models of SE are being used to study the transition of a single episode of SE into chronic epilepsy; the mechanisms of neuronal injury and susceptibility; synaptic reorganization (sprouting); the hippocampal sclerosis; the seizure-induced changes in gene expression and neurogenesis; and the development of new anticonvulsant drugs. Chemical induction of SE in rodents similarly results in the progressive loss of benzodiazepine efficacy [29,30]. In addition, SE is the most widely used approach for inducing chronic epilepsy, especially TLE in rats and mice [31–34]. Unlike acute seizure models, in TLE models, animals exhibit spontaneous seizures without any provocation. The SE episode is generally considered as a trigger to initiate the epileptogenesis in TLE (Figure 1).

This article provides a brief overview of different models of SE and acute neuronal injury (Table 2). Many forms of epilepsy can be modeled in rodents, with seizures induced by chemical treatment, by electrical stimulation or by genetically induced mutations [3,35–37]. Animal models are extremely helpful for the development of an effective drug for SE and neuronal injury [38]. There are several desired features for an ideal SE model:

- Exhibit appropriate seizure phenotype
- Consistent with the neuropathological features of human SE
- Exhibit appropriate latent period following initial insult
- Show post-SE chronic hyperexcitability and neuronal plasticity
- Express spontaneous seizures following a latent period
- Respond to drug therapy and exhibit resistance to certain anticonvulsants
- Allow rapid screening of novel compounds

The ideal animal model of SE should reflect a pathophysiology similar to those of human SE [34,39,40]. Because human SE is a complex neurological disorder that encompasses many causes and seizure phenotypes, it is highly unlikely that any one animal model will truly recapitulate the full spectrum of SE features. Therefore, it is necessary to screen the test compounds in a battery of animal models and also in refractory SE paradigms.

## 2. Electrical Stimulation Models of SE

Electrical stimulation models are the first series of paradigms advanced to study seizures, SE and neuronal excitability. The electrical stimulation models of SE include perforant pathway stimulation and self-sustaining limbic stimulation models.

### 2.1. Perforant Path Stimulation Model

The perforant path stimulation (PPS) approach is widely used for induction of persistent seizures in rats and was pioneered by Sloviter [24,41,97,98]. In this model anesthetized rats receive intermittent, unilateral perforant path stimulation for 24 h. Repetitive tetanic stimulation of hippocampal afferents such as the perforant path [44,98–102], hippocampus [45,103–105], or amygdala [42,104,106] have been used to induce SE and a detailed description of this model is covered elsewhere [38].

#### 2.1.1. Methodology

Rats are stimulated with 0.2 to 0.4 millisecond monophasic rectangular pulses at 20-Hz with 10 s train duration and 30 s intertrain interval through chronically implanted electrodes in angular bundles or fimbria [44]. Electrical stimulation is stopped when 10 consecutive trains each produced 30 s of hippocampal afterdischarge (AD). Rats are then monitored for self-sustained EEG seizure activity; 85% exhibited SE within 7 h. Sloviter described detailed changes following PPS stimulation [98]. Behavioral seizures are rated according to Racine’s scale [104]: stage 0, no response or behavior arrest; stage 1, chewing or facial twitches; stage 2, chewing and head nodding or wet dog shakes; stage 3, unilateral forelimb clonus; stage 4, bilateral forelimb clonus and rearing; stage 5, bilateral forelimb clonus, rearing and falling.

#### 2.1.2. Model Features

Unilateral sustained PPS in anesthetized rat causes necrosis of CA1 and CA3 pyramidal cells and hilar neurons, whereas CA2 neurons are generally unaffected [98]. Unilateral or bilateral neuronal damage was evident following unilateral or bilateral stimulation evoked granule cell discharges, respectively [42]. In addition to the hippocampal lesions, electrical stimulation of the amygdala causes neuronal necrosis in the piriform cortex [99]. Electrical stimulation of the angular bundle can cause extensive aberrant mossy fiber (MF) sprouting in the inner molecular layer of the dentate gyrus [107].

#### 2.1.3. Pros and Cons

The advantage of PPS model is that hippocampal SE can be induced without the complications of direct excitotoxic damage that results from the KA or pilocarpine models. PPS results in a limited lesion in the dorsal hippocampus. Depending upon the area of the brain and the intensity of pulse train electrical stimuli, these models show minor variations in the site and the severity of brain lesions. However, all protocols are generally successful in producing the hippocampal lesions. Histopathologic findings are comparable to those in KA and pilocarpine models, but neurodegeneration is relatively less as compared to KA and pilocarpine models. However, electrode implantation is cumbersome and labor intensive, which reduces the desirability of this model relative to chemoconvulsant models. PPS is a very complex electrical seizure model, which requires sophisticated stimulating-recording equipment. It is primarily used for unilateral or bilateral stimulation to induce SE in rats.

### 2.2. Self-Sustaining Stimulation Model

The self-sustaining limbic (SSL) SE model was advanced by Lothman [45] and other groups [44,46]. Lothman (1989) developed one version of this model of SE, which is centered in the limbic system and elicited by continuous focal electrical stimulation of the hippocampus [45]. The other variations include ventral hippocampal stimulation [46] and hippocampal kindled or naive animals [44]. In the SSL model, a standardized amount of electrical stimulation is delivered to each rat. Under appropriate conditions, the SE persists for many hours after discontinuing the electrical stimulus. The critical determinant for the establishment of the SE is the length of stimulation, rather than the side (left *vs.* right) of stimulation or kindling before stimulation. It shares some similarities with the PPS model and all variations of this model induce SE without direct excitotoxic effects as seen in KA or pilocarpine. A detailed description of this model is described previously [38].

#### 2.2.1. Methodology

Rats are implanted surgically with stimulating electrodes. One week after the surgery, the AD threshold is determined with a standard stimulus of a 10 s train (50 Hz, 1 ms pulse width, square waves). Only rats with an AD threshold less than 250 μA are used. Animals are given stimulation consisting of continuous electrical stimulation delivered to the hippocampus (50 Hz, 1 ms pulses, 400 μA). An individual stimulus epoch lasts 10 min consisting of 10 s on and 1 s off for 9 min. Stimulation is stopped at the 10^th^ min and repeated 9 times. The total duration of stimulation is 90 min. All animals that exhibit SE for 6 to 12 h are classified as SSL-SE. Some animals fail to exhibit SE, which are categorized as non-SSL-SE group [38].

#### 2.2.2. Model Features

Behavioral and electrographic SE features are analyzed in each rat. Mild limbic seizures are equivalent to kindled seizure stages 1 and 2, while severe seizures are equivalent to kindled seizure stages 3 to 5 [104]. Phenobarbital and diazepam have been shown to suppress behavioral seizure activity when given 2 h post SE [108]. However, phenytoin is ineffective in this model. Animals exhibit cell loss in the hippocampus similar to chemoconvulsant models. Bilateral cell loss is observed in CA1 and dentate hilar region [109,110]. Spontaneous seizures are evident in these animals that are similar to complex partial seizures [111]. Self-sustaining SE induced by high intensity (700 μA) electrical stimulation of the basolateral amygdala produced neuronal necrosis in the ipsilateral amygdala, piriform cortex, entorrhinal cortex, endopiriform nucleus, and mediodorsal thalamus in rats. Aberrant mossy fiber sprouting was evident in the inner molecular layer of the dentate gyrus [112].

#### 2.2.3. Pros and Cons

The advantage of SSL model is that sustained SE can be induced without the complications of direct excitotoxic damage that results from the KA or pilocarpine models. The technical feasibility issues including labor and animal care are similar to PPS model.

## 3. Pharmacological Models of SE

There are several chemicals (chemoconvulsants) that are commonly used to induce SE in rodent species. The SE induced by the chemoconvulsants kainic acid, pilocarpine, lithium-pilocarpine and diisopropylfluorophosphate are widely employed to characterize the pathophysiology and to evaluate therapeutic interventions. Glutamate is the primary excitatory neurotransmitter and GABA is the inhibitory neurotransmitter in the brain. Chemoconvulsants that enhance glutamatergic neurotransmission or block GABAergic inhibition are able to induce seizures or SE, while enhancing cholinergic neurotransmission can also trigger seizures by cholinergic hyperactivation.

### 3.1. Kainic Acid Model

Kainic acid (KA) is a prototype agonist of kainate glutamate receptors with potent convulsant activity. KA model of SE is one of the most extensively studied seizure models. Ben-Ari [25] proposed KA-induced seizures as a model with particular relevance to TLE as it shares many of the features of human TLE. KA is a neurotoxic extract of the seaweed *Digenea simplex* [113]. It is a powerful neuronal excitant with high affinity for kainate glutamate receptors [114].

#### 3.1.1. Methodology

Animals are implanted with two surface electrodes (cortex and cerebellum) and a depth electrode in the hippocampus for continuous EEG recording. In rats, intracerebroventricular (0.4–0.8 μg) or systemic administration (8–12 mg/kg, s.c. or i.p.) of KA induces convulsions and progressively developing SE, coupled to epileptiform discharges originating in limbic structures and propagated to other brain areas [47]. In mice, the convulsant dose of KA (20–40 mg/kg, i.p.) is somewhat variable depending on the strain. Following i.p. KA injections, rats display convulsive behavior starting with wet-dog shakes, staring, searching and gnawing, leading to hyperactivity, forelimb clonus and tonic-clonic convulsions [48]. The behavioral seizure activity is generally rated according to Racine’s scale [104]. Wet dog shakes, head nodding and facial clonus are given a seizure score of stage 1 to 2, forelimb clonus is stage 3, rearing is stage 4 and continued rearing and falling is stage 5. During the first hour after KA injections there are changes in the behavior that include staring episodes followed by head bobbing and numerous wet dog shakes. This is followed by isolated limbic motor seizures that increase in frequency, eventually leading to SE. Similar behavioral reactions in rats and mice were reported following ICV injections of KA. KA induced SE mimics human SE in progressive, sequential EEG changes. In order to get consistent SE and lesion accompanied by MF sprouting and spontaneous seizures, a multiple KA injection method was developed [31,92]. Adult rats are given KA (5 mg/kg, i.p.) once per hour until continuous SE develops. Repeated stage 4 and 5 seizures occur over a 4–6 h period, and the survival rate is better than through a single large dose protocol.

#### 3.1.2. Model Features

KA induced SE results in extensive damage in the following brain regions: hippocampus, amygdala, piriform cortex, entorrhinal cortex, septum and medial thalamus. Damage in the hippocampus includes CA1 and CA3 pyramidal neurons and hilar neurons in the dentate gyrus [31]. The CA2 pyramidal neurons and dentate granule cells appear to be resistant to damage induced by KA. The pattern of neuronal loss after KA-induced SE is symmetrical, that is, bilateral structures exhibit the same degree of cell death. However, the pattern of damage is often variable among rats receiving the same treatment [31,43,47,49–51]. SE induced by KA later leads to the development of spontaneous limbic seizures and MF sprouting in the dentate gyrus of the hippocampus. This sprouting is similar to that observed in human hippocampal tissue from cryptogenic epileptic patients [52,115,116]. New axon fibers from the granule cells grow into the inner molecular layer of the dentate. It has been suggested that the new pathway is functional and proconvulsant, synapsing on the dendrites of the granule cells, thereby creating a recurrent excitatory pathway [4,117].

#### 3.1.3. Pros and Cons

KA produces robust and persistent seizures associated with neuronal damage similarly found in human epileptogenic tissue. It is very simple to use and does not require sophisticated equipment, except for monitoring of EEG. Regardless of the route of administration, KA induces SE that electrographically resembles SE in humans [118]. A major drawback of the KA model is the variable sensitivity of rats of different strains, sex, age and weight to KA [47]. Aged rats exhibit SE at lower doses of KA with greater neuronal damage [119,120]. The other limitation of KA model is the direct excitotoxic action of KA that makes it difficult to separate direct neuronal damage from seizure-induced neuronal damage [31]. However, it is a less than ideal model for testing anticonvulsants for SE because not all of the currently effective drugs are effective in the KA model.

### 3.2. Pilocarpine Model

The pilocarpine model of SE is one of the well-established animal models for SE and shares many of the characteristics of human TLE [121]. Turski first reported that pilocarpine, a muscarinic cholinergic agonist, induces robust limbic seizures when systemically administered to rats (400 mg/kg) and mice (300–350 mg/kg) [27]. They observed a sequence of behavioral alterations including staring spells, limbic gustatory automatisms, and motor limbic seizures that overtime (1–2 h) progressively developed into limbic SE that last for several hours [53,54]. The pilocarpine is given via intraamygdaloid [55], intrahippocampal [56] or systemically by intraperitoneal injections [27,57]. Pilocarpine induced changes in EEG activity first appear in the hippocampus followed by the amygdala and neocortex, but later it was shown that initial EEG alterations occur in ventral forebrain [26]. This may explain the absence of wet dog shakes at the beginning of pilocarpine-induced SE. Within 24 h of pilocarpine injection, the seizures subside and the EEG returns to normal activity [27]. Pretreatment with scopolamine and diazepam are able to prevent the pilocarpine-induced seizures [53]. This model allows for studying the generation and spread of convulsive activity in the hippocampus and amygdala. The initiation of the seizures is due to activation of cholinergic system since pretreatment with atropine prevents the seizure occurrence. It is thought that pilocarpine initiates SE by cholinergic hyperactivation, but that continuation of seizure activity is likely through a glutamatergic mechanism. Neuronal loss and spontaneous seizure activity occurs secondary to seizure-induced glutamate release [26,59].

#### 3.2.1. Methodology

For continuous EEG recording, two surface electrodes (cortex and cerebellum) and a depth electrode in the hippocampus are implanted in the rat or mice under anesthesia. After a recovery period of 1–2 weeks, the animals are given pilocarpine injections (rats, 400 mg/kg, i.p.; mice, 300–350 mg/kg, i.p.). The animals are pretreated with scopolamine methylnitrate (1 mg/kg), a muscarinic antagonist by s.c. injection to prevent the peripheral effects of pilocarpine. Thirty minutes later pilocarpine hydrochloride (300–400 mg/kg) dissolved in saline is injected intraperitoneally. Limbic motor seizures are initiated by pilocarpine within 30 min of the injection. The latency from the time of the pilocarpine injection until the onset of behavioral seizures and SE appears to be dose dependent [58]. With higher doses more animals exhibit SE, but this is accompanied by an increase in mortality.

#### 3.2.2. Model Features

Injections of large doses of pilocarpine induce EEG and behavioral limbic seizures and SE. Pilocarpine-induced SE causes massive neuronal damage when examined at 24 to 72 h. Studies have found cell death in olfactory cortex, the amygdaloid complex, thalamus, neocortex, hippocampus and substantia nigra [27,55–57,122]. The extensive brain damage, characterized by shrunken neuronal cell bodies with edematous neurophil, is present in the anterior olfactory, piriform and entorhinal cortex. The basal amygdala and ventral hippocampus are very sensitive to pilocarpine-induced SE. The majority of the damage in the dorsal hippocampus occurs in CA3 and CA1. Neocortical cell loss occurs mostly in layer 2 and 3, with some cell loss in layer 5. The par reticulata of the substantia nigra is extensively damaged in this model [26]. Loss of hilar interneurons is a hallmark in the pilocarpine model of SE [123–125]. SE induced by pilocarpine later leads to the development of spontaneous limbic seizures and MF sprouting in the dentate gyrus of the hippocampus.

#### 3.2.3. Pros and Cons

Pilocarpine is a powerful cholinergic agent and produces persistent seizures associated with neurodegeneration. It is very simple to use and require little sophisticated equipment. Pilocarpine is generally used for systemic administration to induce SE in rats and mice. The SE induced by pilocarpine is similar to SE induced by kainic acid and the site of initial EEG changes in the two models is different. The pattern of neuronal damage is similar to KA model but pilocarpine induces greater neocortical damage [126]. As in the KA model, the pilocarpine-induced SE leads to the development of spontaneous limbic motor seizures and MF sprouting in the dentate gyrus [127]. Since hippocampal synaptic reorganization and MF sprouting is a common feature of human epileptogenic tissue [115,116,128], the pilocarpine model is often used to examine the relationship between MF synaptic reorganization and spontaneous seizure activity [127,129].

### 3.3. Lithium-Pilocarpine Combination Model

Pilocarpine alone at a high dose of 400 mg/kg (i.p. or s.c.) may not induce consistent SE. In order to enhance the action of pilocarpine, a small amount of lithium chloride (LiCl; 3 mEq/kg, i.p.) is given to rats 19 to 24 h prior to the administration of pilocarpine. This results in a significantly lower dose of pilocarpine (20–30 mg/kg) needed to induce SE. The progression of continual seizures that occurs can be considered as generalized convulsive SE [130]. Pretreatment with LiCl seems to potentiate the effect of pilocarpine, since lithium in combination with a low dose of pilocarpine induces most consistent SE in rats [26,60,122]. All other features are similar to SE induced by high doses of pilocarpine.

#### 3.3.1. Methodology

Rats are implanted with two surface electrodes (cortex and cerebellum) and a depth electrode in the hippocampus for continuous EEG recording. Persistent SE is chemically induced by lithium-pilocarpine in adult rats [90]. Briefly, rats are injected with two doses of pilocarpine (20 mg/kg, i.p., per 30 min) to induce persistent SE. Lithium chloride (3 mEq/kg, i.p.) treatment is given 18–24 h prior to pilocarpine. Scopolamine (1 mg/kg, s.c.) is given 30 min prior to pilocarpine to counteract peripheral side effects of pilocarpine. The onset and termination of SE are determined by behavioral seizures and EEG recordings from the hippocampus and the cortex. Animals are perfused at different time points after SE induction for immunohistochemical studies.

#### 3.3.2. Features

The electrographic and behavioral features of the lithium-pilocarpine regimen are almost identical to the pilocarpine model. Treated animals exhibit electrographic and behavioral SE for over 5 h, which represent a state of refractory SE. Moreover, these seizures respond to benzodiazepines when given early, but become resistant with time. The extent of neuronal injury is comparable to pilocarpine or other chemical models of SE.

#### 3.3.3. Pros and Cons

Lithium-pilocarpine induced SE shares similar time characteristics for onset and duration with the high-dose pilocarpine model, although they differ starkly in onset severity. The mortality rate in the lithium-pilocarpine model is very low compared to the high dose pilocarpine model.

### 3.4. Organophosphate Pesticide Model

Organophosphate (OP) pesticides are able to induce sustained SE in rodents. OP pesticides such as diisopropylfluorophosphate (DFP) and paraoxon are highly neurotoxic agents [131,132]. When exposed deliberately, or by accident or natural disaster, they cause SE and neuronal damage quite similar to that of nerve agents. Benzodiazepines such as diazepam and imidazenil protect against DFP-induced seizures when given early after exposure [61,95]. Recently, the DFP model of SE was characterized in rats [62,63]. DFP is very similar in structure to the nerve agents soman and sarin. The OP pesticides and nerve agents are extremely lethal and produce neurotoxicity via common mechanisms [133–135]. They cause devastating damage to the brain primarily due to their irreversible inhibition of acetylcholinesterase, leading to an excessive accumulation of acetylcholine, a powerful excitatory neurotransmitter in the brain. Exposure to OP intoxication results in cholinergic hyperactivation and causes a set of predictable toxic signs: hypersecretion, fasciculations, tremor, convulsions, respiratory distress and death. CNS manifestations following OP exposure include convulsive seizures and SE, which can last 30 min or longer with profound brain damage, resulting in death, or long-term neuronal damage. The effects of OP intoxication are very long lasting and survivors suffer chronic brain damage such as risk of epilepsy and cognitive deficits.

#### 3.4.1. Methodology

The procedure involves systematically administered DFP-induced persistent seizures and SE in rats [37,62,63]. Animals should be pretreated with pyridostigmine bromide (0.026 mg/kg, i.m.) 30 min before DFP injection. One minute following DFP injection (1–4 mg/kg, s.c.), animals should be given pralidoxime chloride (2-PAM, 25 mg/kg, i.m.) and an atropine injection (2 mg/kg, i.p.). This increases survival rates without affecting the severity of seizures, since atropine and 2-PAM do not cross the blood-brain barrier. Rats are allowed to undergo convulsions and SE-like activity following DFP. Behavioral and EEG activity are monitored to assess the seizure activity. When animals exhibit at least 10 min of seizure activity, they will be injected with test drugs. Behavioral and EEG seizure activity are recorded for 24 h by a video-EEG system. DFP intoxication seizures are rated as per the modified Racine scale: stage 1, chewing or excess secretion (SLUD, salivation, lacrimation, urination, and defecation); stage 2, whole body twitching/tremors; stage 3, unilateral forelimb clonus or paralysis; stage 4, bilateral forelimb clonus; stage 5, severe convulsions; and stage 6, death.

#### 3.4.2. Pros and Cons

OP agents produces SE in a similar fashion to pilocarpine. However, their mechanism of action is quite different from pilocarpine. OP pesticides are irreversible acetylcholinesterase inhibitors while pilocarpine acts as agonist at muscarinic cholinergic receptors. OP pesticides are very potent and difficult to handle in the lab. Moreover, there are few effective antidotes for OP intoxication, especially for rapid termination of seizures, SE and brain damage. Current approved treatment for OP intoxication or nerve agent exposure is a specialized drug combination containing; (i) atropine—a muscarinic receptor antagonist; (ii) pralidoxime—a drug to regenerate acetylcholinesterase activity; and (iii) diazepam—a benzodiazepine to reduce seizures [61,134]. Diazepam treatment must be administered within 40 min, after which there is no protection against seizures and progressive neurological damage occurs. High doses of diazepam are needed to control recurrent seizures, resulting in sedation, respiratory depression and tolerance. Seizures induced by OP intoxication can become self-sustained and develop time-dependent refractory SE—a serious condition with significant brain injury and mortality. Benzodiazepines are not highly effective against refractory SE that occurs at later times after OP exposure [93]. The DFP is an important chemical model that replicates several features of refractory SE and could be useful for testing novel AEDs, as the current treatment is not always successful when given late after seizure onset.

### 3.5. Flurothyl Model

Flurothyl is a volatile liquid that belongs to the halogenated ether family. It has stimulant and convulsant properties [64,65]. It was previously used in psychiatric medicine for shock therapy, but such use has now been discontinued. Flurothyl is widely used in experimental epilepsy research for inducing seizures in animals. Flurothyl is used mainly for acute testing of seizure threshold or to sustain the seizures during the flourothyl exposure period. Rats are observed for behavioral seizures following exposure to inhalational flurothyl. Flurothyl seizures are induced in an airtight chamber (volume 9.4 L). The animal is placed in the chamber and flurothyl delivery is initiated at a constant rate of 40 μL per min onto a pad of filter paper [66,67]. Flurothyl (SynQuest Laboratories, Inc., Alachua, FL) is liquid convulsant ether with a low boiling point temperature. Therefore, it easily evaporates from the filter paper and is inhaled by the tested rat. Flurothyl induces two different primarily generalized seizures: clonic seizures of face and forelimbs with preserved righting ability, and tonic-clonic seizures of all four limbs after the righting ability has been lost. It has been determined that in immature rats, flurothyl-induced clonic and tonic-clonic seizures develop in very fast succession [67]. From collected data, thresholds can be calculated for seizure types in terms of amount of infused flurothyl required for the induction of that particular seizure and latency to onset of clonic and tonic-clonic seizures [136]. Continued administration of flurothyl results in prolonged seizure and SE. Unlike pentylenetetrazol or other chemoconvulsants, flourothyl is a very volatile compound and easy to administer by inhalation techniques. This is a rapid test for determination of seizure threshold in rodents. It is also used to assess the seizure susceptibility of genetically-altered mouse models.

### 3.6. Cobalt-Homocysteine Model

The cobalt-homocysteine model of SE was pioneered by Walton and Treiman in 1988. Generalized convulsive SE can be induced in rats with cortical cobalt lesions when challenged with d,l-homocysteine thiolactone (HCTL) by intraperitoneal injection [68]. This induced focal motor seizures that secondarily generalized, providing a unique model of SE. An active epileptic focus is induced with application of cobalt powder in the left anterior lobe, through holes drilled in the skull while the rat is under anesthesia. Animals are allowed to recover at least 4 days, at which time EEG activity is monitored daily. After the occurrence of behavioral and electrographic seizure activity, each animal is injected with HCTL (5.5 mmol/kg, i.p.). The average time from placement of cobalt until injection of homocysteine thiolactone is 9 days. Seizures occur within 10 to 15 min after the HCTL and secondarily generalized tonic-clonic seizures are observed within 30 min of the HCTL injection. EEG patterns observed during SE are very similar to those seen during human SE. Phenytoin, phenobarbital, diazepam and lorazepam are effective in terminating the generalized seizures when given i.p., after the second such seizure. This profile is predictive of their efficacy in the treatment of human SE [137]. However, the model has significant limitations. First, the neuropathology associated with SE has not been characterized in this model. Second, there is significant mortality during SE in this model. Finally, it is a tedious model with a great deal of preparation time for cobalt lesion followed by HCTL challenge.

## 4. Thermal Models

Thermal models mostly consist of increasing core body temperature or eliciting febrile (fever) seizures in rodents. Febrile seizures are the most common in children under 5 years of age. Febrile seizures are simple or complex depending on the severity and duration of seizures. Febrile seizures that are prolonged reaching SE or recurrent within a given fever are referred as “complex febrile seizures”. Such prolonged febrile seizures occur in a fraction of children and such children are at high risk for later acquired epilepsy [138,139]. Given the emerging data suggesting that longer febrile seizures are more likely to lead to epilepsy, there is increasing interest in developing febrile seizure models of SE and neuronal injury.

### 4.1. Hyperthermia (Complex Febrile) Model

There has been a great deal of effort to develop a practical and clinically relevant animal model of febrile seizures. There are numerous obstacles to develop and validate a realistic model. Because febrile seizures are provoked by fever, the idea is to induce fever-like conditions in rodents. However, it is not easy to provoke fever in young rodents during the developmental ages that correlate with human childhood. Consequently, many complex approaches have been utilized to model the fever in rodents. In one model, rats are treated with the bacterial endotoxin lipopolysaccharide, which causes an immune response and augments core temperature in immature rodents by about 1 °C. Seizures are then evoked by a chemoconvulsant [140]. The most widely employed method has been to raise the core temperature by heating the animal [69–72]. Over the years, a variety of heating methods has been used, including hot water, infrared heat lamp, and warmed air stream. In these models, the animal’s core temperature rises and consequently, brain temperature also rises and this leads to hyperthermic seizures [71]. Although hyperthermic seizures represent febrile seizures, such seizures are not true febrile seizures, which are triggered by endogenous pyrogenic mediators. Nevertheless, febrile seizures models can provide valuable insights on the mechanisms underlying the injury and the long-term impact on neurological functions.

#### 4.1.1. Methodology

Baram and colleagues developed a rat model in which seizures are induced by an external heat stream using a hair dryer [69,70,72,73]. Febrile seizures are induced in rat pups at P11. Two rats are placed in a glass jar and were subjected to a regulated airstream (39.5–41 °C) to raise their core temperature. Core temperatures are measured at baseline, at seizure onset (an average of 2.9 min from the onset of hyperthermia) [69] and every 2 min during the seizures, and were maintained by moving rats that were close to the higher end of this range to cool surfaces for 2 min. There are two protocols with varied duration: 30 or 70 min. The behavioral seizures induced by the hyperthermia have previously been correlated with EEG seizures [73] and are stereotyped, consisting of sudden movement arrest, followed by facial automatisms (chewing) and forelimb clonus, which might evolve to body flexion with biting of an extremity and rarely to generalized (tonic) seizures. After 70 min of hyperthermia, animals are weighed and moved to a cool surface until core temperature is reduced to the normal range for age (34–35 °C), and then returned to the dams.

#### 4.1.2. Pros and Cons

Hyperthermic models are simple paradigms for febrile seizures. Prolonged hyperthermia may mimic the complex febrile seizures including the risk for epileptogenesis. Seizures in this model occur reliably when core temperature reaches 39.5 to 41 °C, usually within 3 min of hyperthermia, and involve fever mediators. The seizures are remarkably reproducible and exhibit characteristics that suggest limbic origin, involving behavioral arrest, staring, chewing and other facial automatisms, and forepaw clonus. Like human febrile seizures, rats experiencing hyperthermic seizures have elevated levels of the interleukin IL-1β, and show interictal discharges representing hyperexcitability. However, no neuronal death is observed in these models [74]. However, fever in children is an endogenous phenomenon due to pyrogenic substances. In rodents, it is induced with forceful heating of the body. The consequent heat injury could be a major confounder in these models. Moreover, there is debate about the duration of hyperthermia state or seizures. In children, a febrile seizure of either 24 min or 64 min would be considered complicated, while the 64-min seizures qualify as febrile SE for modeling considerations. Epileptogenesis is observed in a significant proportion of rats experiencing post-hyperthermia SE events with little neuronal loss. This phenomenon is used to exemplify that neuronal death is not necessary for acquired epileptogenesis in the immature brain [141].

## 5. *In Vitro* Models of SE

*In vitro* techniques such as electrophysiological approaches are commonly used for elucidating the mechanism of action of anticonvulsants. The most popular and widely employed electrophysiological approaches include hippocampus slice electrophysiology and patch-clamp in single neurons. The most direct evidence support a specific mechanism at the receptor or ion channel level is obtained from patch-clamp electrophysiology. Although time-consuming and labor intensive, electrophysiology can provide extremely valuable information on the ability of a drug to modify a receptor-gated or voltage-dependent ion channel. Electrophysiological recordings are often conducted using acutely dissociated neurons, neurons maintained in primary culture, or neurons in an acutely isolated brain slice. Different recording configurations are used (e.g., whole-cell, cell-free, and nucleated patches) to examine macroscopic or single-channel activity. Brain slices are the most convenient preparations for *in vitro* studies. The neuronal circuitry inherent in the slices makes it suitable for the study of epileptiform activity under both control and hyperexcitable conditions. Extracellular recording techniques using isolated brain slices can provide useful information pertaining to the effect of an AED on population events and evoked or spontaneous epileptiform bursting, depending on whether the slice is obtained from a healthy or epileptic animal. Spontaneous epileptiform bursting in epileptic animals can be studied using extracellular recordings. Epileptiform bursting can be evoked by perfusion of hippocampus slices with ion channel blockers such as 4-aminopyridine, bicuculline or picrotoxin [81–83,142–146]. Low magnesium ACSF is the most widely used approach for eliciting spontaneous epileptiform bursting in the hippocampal slices. Test compounds can be applied in the perfusion and their ability to reduce the spontaneous epileptiform bursting or evoked activity provides a powerful index of drug’s efficacy to reduce seizure activity.

There are numerous advantages with brain slice preparation: (i) it has well characterized neuronal circuitry; (ii) anatomical boundaries are easily visualized under microscope; (iii) absence of input from the remainder of the brain structures; (iv) precise electrode placement for recording; (v) rapid alteration of activity with fast exchange of drug solutions; and (vi) provides the convenience of onset and offset of changes in activity. Moreover, this technique avoids the inherent problems associated with penetration through the blood brain barrier in animal models. However, there are several limitations with this technique. There is significant difference in the response due to lack of endogenous or circulating factors, absence of input from the remainder of the brain, and greater variability in response depending on age and time-course of recording in the slice.

### 5.1. Low Magnesium Model in Slices

Low or zero-Mg^2+^ is a commonly used *in vitro* model of epileptiform activity in entorrhinal-hippocampal slices used to determine the effect of drugs on seizure activity and mechanisms of antiepileptiform activity [75,76,147,148]. The zero-Mg^2+^ model in horizontal entorrhinal-hippocampal slices is of particular importance: epileptiform activity is progressive and develops resistance to benzodiazepines after prolonged periods [75,149,150]. Horizontal slices containing key cortical areas and the hippocampus are isolated from neonatal or young mice or rats [77]. The zero-Mg^2+^ model produces synchronized epileptiform activity in hippocampal slices, as assayed by extracellular field potential recordings in several temporal lobe areas, including the entorrhinal cortex and CA1 areas [149]. The epileptiform activity progresses through two stages: the first stage is characterized by seizure-like events that are sensitive to benzodiazepines, and the second stage occurs after nearly 1 h, and is characterized by an abrupt change to late recurrent discharges that are insensitive to benzodiazepines [75,149]. Epileptiform activity is assessed through the frequencies, durations, and amplitudes of seizure-like events and late recurrent discharges throughout the recording period. Drug efficacy is determined by a complete cessation of the field potentials [75]. These parameters are also plotted against time as a measure of the temporal development of drug-resistant epileptiform activity.

### 5.2. High Potassium Model in Slices

Epileptiform activity can be evoked with high [K^+^]_o_ ACSF perfusion of hippocampal slices [78–80]. Preliminary studies of the concentration-response of K^+^ concentration in the ACSF are needed to determine the K^+^ levels that are below and above threshold for generation of paroxysmal depolarization shifts (PDSs) in CA1 pyramidal neurons. In many hippocampus preparations, 7 mM [K^+^]_o_ ACSF is consistently threshold for evoking PDSs for several applications. Elevated [K^+^]_o_ seizure models are particularly relevant to studies of astrocytes, which play a key role in buffering extracellular K^+^ affecting neuronal excitability.

### 5.3. 4-Aminopyridine Model in Slices

4-Aminopyridine (4-AP) is a potent convulsant. 4-AP acts as a blocker of K^+^ currents, mainly of the early transient K^+^ current and acts on presynaptic targets leading to enhancement of neurotransmitter release at both inhibitory and excitatory synapses, including release of glutamate. When applied or injected in low concentrations, 4-AP readily evokes epileptiform activity in both *in vivo* and *in vitro* models [142–144]. The convulsant properties of 4-AP have also been reported clinically in humans [81]. Unlike other convulsant drugs that act primarily by diminishing the efficiency of GABA-mediated inhibition [84], the evidence available indicates that 4-AP-induced epileptiform discharges occur despite the presence of normal or even enhanced synaptic inhibition [144,151,152]. Hence, 4-AP is thought to be a suitable model to investigate the pathophysiological mechanisms involved in the generation of epileptiform activity in conditions where synaptic inhibition is preserved. However, the mechanisms underlying the 4-AP’s seizure induction remains unclear.

4-AP is the most popular *in vitro* method for testing compounds on epileptic seizures because of its potent and reproducible induction of epileptiform bursting in slices [83,84,151–153]. In this model, extracellular recordings are made from the CA3 region using glass microelectrodes filled with 2 M NaCl (resistance, 2–10 mΩ). After equilibration of slice, an insulated bipolar stimulating electrode is positioned in the dentate gyrus mossy fibers, and a glass microelectrode filled with ACSF is positioned in the stratum pyramidale using an optical microscope. The position of the recording electrode can be adjusted to obtain a maximal amplitude signal. This is optimized by recording population responses that were evoked with a bipolar stimulating electrode via a constant-current isolation unit at an intensity of 200–350 μA for 100 μs. 4-AP (75 μM) is bath applied at a rate of 2–3 mL/min for induction of spontaneous epileptiform bursting [82,83]. Perfusion of ACSF containing 4-AP (75 μM) elicits typical high frequency epileptiform bursting (~30 events/min) recorded from area CA3. The spontaneous bursting typically starts within 5 min of the onset of 4-AP perfusion and increases gradually to a plateau level at about 30 min. Bath washing of slices with ACSF free of 4-AP leads to a gradual disappearance of bursting. In this preparation, test drugs can be perfused simultaneously with 4-AP to check their ability to suppress spontaneous bursting. The inhibitory effect of test compounds is observed for the 60 min recording period. The slice model provides important insights about the cellular mechanism of anticonvulsant agents under conditions that avoid confounding factors such as differences in absorption, metabolism, and brain accessibility.

### 5.4. Organotypic Slice Culture Model

Acute brain slices are prepared and used on the same day. Organotypic slice techniques are introduced to allow for the use of brain slices in prolonged culture. Brain slices from young rodents can be maintained in culture for many weeks to months [85–89]. Such organotypic slices are prepared in much the same way as acute slices but the tissue is usually taken from neonatal animals. They are generally prepared according to the interface culture method from 8 to 11-day-old rats. They are maintained by culturing at an air/liquid interface, either by continuously rotating the preparation (roller-tube cultures) or by culturing them on semiporous membranes (stationary interface cultures). The basic requirements include a stable substratum, culture medium, sufficient oxygenation and incubation at a temperature of about 36 °C. Under these conditions, nerve cells continue to differentiate and to develop a tissue organization that closely resembles that observed *in situ*. Slice cultures are useful for a variety of applications including induction of SE.

Organotypic hippocampal slices are commonly used to study epileptiform discharges and neuronal injury. They have several advantages over acute slices. Drugs can be applied in known concentrations and removed when desired. Due to their high neuronal connectivity, slice cultures provide a very useful tool for studying the properties of synaptic transmission between monosynaptically coupled cell pairs. It provides a convenient *in vitro* system to study paired recordings between two neurons in a slice. Application of chemical substances or excitotoxins to slice cultures can be used to examine their short-term or long-term consequences on epileptic discharges, as well as the effects of test compounds to prevent or block the neuronal abnormality including seizure activity [154–158]. The slices tend to thin out in culture, providing an optically advantageous preparation. Organotypic brain slice cultures are also used as a medium throughput, *in vitro* neuroprotection assay for studies of neuroprotection following SE and other brain insults. By using fluorescent compounds that binds to the DNA of injured or dying neurons, such as propidium iodide, neuronal cell death can be analyzed in slice cultures following application of chemoconvulsants such as KA or glutamate receptor agonists. Organotypic slices have many disadvantages. They are more difficult to prepare and maintain than acute slices, and it has not been feasible to make such slice cultures from adult brains. It is generally recognized that the synaptic organization is not exactly the same as in native brain tissue. Moreover, many chemoconvulsants do not produce excitotoxicity in organotypic hippocampal slices. This limits its utility in seizure-induced neuronal injury and neuroprotection studies.

## 6. Refractory SE Models

Benzodiazepines are the drugs of choice for the treatment of SE. However, 35%–50% patients exhibit partial or complete resistance to standard drugs, a condition known as refractory SE. Newer anticonvulsants are needed for rapid and effective termination of refractory SE. Key features of refractory SE can be observed in experimental models. Walton and Treiman first observed drug refractoriness in an experimental model of SE [159]. In refractory models, SE is characterized by persistent seizures, progressive internalization of GABA-A receptors, and benzodiazepine resistance [93,94]. However, there is little information on the comparative analysis of animal models that recapitulates neurological features of refractory SE [160]. There are several models that have been well characterized to represent the refractory SE by determining diazepam’s resistance to abort seizures and neurodegeneration. The protracted seizures caused by pilocarpine, KA and DFP are widely employed to model refractory SE in rodents. There is evidence for time-dependent phenytoin’s resistance in an electrical stimulation model of SE [161].

### 6.1. Pilocarpine Model

The pilocarpine with or without lithium priming is widely used as a model of human SE because it reproduces many of its features, including refractory seizures, selective interneuron loss, and poor control of seizures by anticonvulsants [37,90,91]. SE is induced by pretreatment with lithium (3 mEq/kg, i.p.) followed 18–24 h later by two pilocarpine (20 mg/kg, i.p.) doses separated by 30 min. Such a regimen produces behavioral seizures and electrographic SE that lasts for >5 h. Rats treated with lithium-pilocarpine exhibit typical behavioral features of cholinergic stimulation and progressive SE such as behavioral arrest, excessive salivation, chewing, facial twitching, unilateral and bilateral forelimb clonus, rearing with or without loss of postural control, and running. EEG recordings are essential to show the electrographic activity during SE, both before and after diazepam treatment. Diazepam (5–10 mg/kg, i.p.) treatment is given at 1 or 2 h after seizure onset because previous reports showed refractoriness to diazepam in a time-dependent fashion, especially at the late time points [30,90]. When administered 40-min or later after the onset of SE, diazepam often fails to reduce the severity of seizures, and seizure recurrences is more common during the course of treatment monitoring. Therefore, the prolonged seizure activity that is not effectively terminated by the late intervention by diazepam certainly represents a state of refractory SE [37]. The main morphological features of acute consequences of pilocarpine treatment include: (a) Hippocampal damage characterized by cell loss in the hilus, CA3, and CA1 regions, (b) The cell loss of CA3 pyramidal cells that leads to an important reduction in the Schaffer collateral input, (c) Cell loss in CA1 affecting specific types of GABAergic interneurons, (d) Cell loss in other limbic structures such as amygdala, but not within the hypothalamus and cortical structures.

### 6.2. KA Model

KA causes persistent seizures that are often referred to as refractory SE. The KA model induces a continuous seizure pattern of SE in mice that results in electrical spiking and tonic-clonic seizures that progressively worsen over the course of several hours [25,31,92]. Benzodiazepines are first-line drugs commonly used to suppress seizures in humans and animal models of SE [93]. However, the therapeutic dosage must be substantially increased after 50 min of continuous seizures in rats [30] and this is similar to the lack of therapeutic efficacy of benzodiazepines after prolonged periods of SE in human patients. Key parameters such as EEG recordings and behavioral seizure ratings are used to demonstrate the drug efficacy in the KA model of refractory SE. The efficacy of drug treatment is measured as the reciprocal of the total number of spike waveforms observed between 5 min after drug administration and the point at which the animals are euthanized. Based on the mortality rates in various studies, investigators use percent survival as another measure of the therapeutic efficacy of test drugs. EEG spike waveforms are routinely used to determine the latency to first seizure, total time spent seizing, the number of seizures and spike waveforms, and the percent survival for each treatment group. Biochemical investigations are performed to determine the mechanisms that underlie seizure progression and pharmacoresistance to benzodiazepines in the KA model of SE.

### 6.3. DFP Model

With the renewed focus on chemical threat agents and pesticide poisoning, DFP has received prominence due to its ability to cause SE, especially refractory SE in rodent models [37,62,95,96]. Persistent SE can be induced by OP intoxication in rats with exposure to DFP, an OP insecticide that, like nerve agents, causes SE and neuronal damage. To increase the survival, atropine sulfate and 2-PAM are administered to mimic available field treatment regimens. Diazepam is administered at 40 or 60 min after the onset of SE to attenuate seizures and ascertain benzodiazepine resistance. The onset and termination of SE are generally determined by video EEG recordings for up to 24 h. DFP-treated animals show electrographic and behavioral SE for over 8 h, which represent a state of refractory SE. Diazepam partially reduces seizures but sustained suppression of either behavioral or electrographic seizure activity is not observed in many animals, a profile indicative of refractoriness. Moreover, at 72 h following SE induction, a massive neuronal death in CA1, CA3 pyramidal regions, and in the dentate hilus regions is evident [37]. The extent of neuronal damage in the DFP model was similar in pilocarpine and KA models. Diazepam produced marginal protection against the SE-induced neurodegeneration in both models [37]. Overall, in the DFP model, persistent seizure activity and neurodegeneration are comparable to pilocarpine model; therefore it may provide a field model of refractory SE that has unique advantages than other models.

## 7. Morphological Approaches

In many animal SE models, cell necrosis and neurodegeneration are hallmark features. Acute neuronal injury and neurodegeneration are routinely assessed to estimate the extent of protection provided by the test compounds. The following methods are used to determine the neuroprotective potential of test compounds.

### 7.1. Cell Necrosis and Apoptosis

#### 7.1.1. Nissl Staining

Histological assessment is made using Nissl (Cresyl violet, CV) staining techniques, as described previously [31,162]. CV stains all neurophil components and any changes in cytoarchitecture of the hippocampal cell layers like loss of cells is determined. Cresyl Violet Acetate solution is used to stain Nissl substance in the cytoplasm of neurons. It stains both neurons and glia and is very useful to identify the overall cell loss and neuronal damage.

#### 7.1.2. TUNEL Assay

To measure apoptotic cell death, terminal deoxynucleotidyl transferase and digoxigenin-11-dUTP nick end labeling (TUNEL) staining is mostly widely employed using the Apoptag peroxidase *in situ* apoptosis detection kit [31]. For quantification of cell death, photomicrographs (30-μm) of three sequential sections are taken at dorsal hippocampus level of each animal. The representative sections from different animals (*n* = 4) are permeabilized by treating with 0.25% trypsin solution in 0.01 N HCl for 30 min at 37 °C and then washed in PBS, and nonspecific sites are blocked by using 0.1 M Tris buffer containing 3% bovine serum albumin and 20% normal bovine serum for 30 min at 37 °C. After this, sections are washed in PBS and incubated in TUNEL reaction mixture for 90 min at 37 °C. The TUNEL reaction mixture comprised enzyme solution and fluorescein label solution at a ratio of 1:9. The sections designated as negative controls were incubated in fluorescein label solution only. The nucleus of apoptotic cells in sections treated with the TUNEL mixture exhibits a clear green fluorescence. TUNEL-positive cells within a square millimeter area are counted by an observer blind to the treatment conditions.

#### 7.1.3. Fluoro-Jade B Staining

For detecting degenerating neurons and their processes, Fluoro-Jade B (FJB) staining is commonly performed in brain sections from rodent models [31,162]. This staining procedure is a sensitive and reliable marker for neuronal degeneration that results from SE or brain injury [163,164]. The extent of neurodegeneration after the SE is determined with FJB staining in dentate hilus (DH), CA3 and CA1 sub-regions of the hippocampus. The rats are perfused with 4% paraformaldehyde solution and the brains collected and processed in 30% sucrose. Consequently, 30 μm thick sections of these brains are cut with cryostat at the dorsal hippocampus level and collected in phosphate buffer solution (PBS) and later stored at −20 °C in cryo-buffer. To visualize neurons undergoing degeneration after SE, three sequential sections each 450 μm apart through the dorsal hippocampi are collected in each animal, mounted on gelatin-coated slides and air dried at room temperature overnight. Slides were then washed sequentially in 100% ethanol, 70% ethanol and deionized water. The slides were then incubated in 0.06% potassium permanganate solution for 15 min (with slow shaking), washed in deionized water, and incubated in 0.004% FJB (Histo-Chem Inc., Jefferson, AK ) dissolved in deionized water with 0.1% acetic acid for 30 min. Then slides were washed three times for 1-min in deionized water, dried on a slide warmer at 55 °C, briefly immersed in xylene and cover slipped using DPX mounting media. The sections are analyzed by confocal microscope using FITC filter. Cells labeled by FJB were detected as individual green shiny pyramidal shaped spots clearly identifiable from background. The number of FJB positive cells (dying neurons) per image field in the hippocampal DH, CA3 and CA1 are counted in each of the three sections per animal [165].

### 7.2. Neurodegeneration, Neurogenesis and Mossy Fiber Sprouting

#### 7.2.1. Neurodegeneration

The extent of neurodegeneration after the SE is determined by the immunostaining of sections for NeuN (principal neurons) and parvalbumin- or neuropeptides-Y-positive (interneurons) cells [31]. The neuronal nuclei antigen (NeuN) is a very specific protein that is highly expressed in nucleus and less expressed in cell body in differentiated neurons. NeuN is not expressed in glial cells, oligodendrocytes, astrocytes, or microglial cells, and cerebellar Purkinje cells. Thus, NeuN immunohistochemistry is commonly used to identify neuronal loss and to quantify total number of neurons in various rat brain regions. To analyze the overall neurodegeneration after the SE, the brains are removed, postfixed in 4% paraformaldehyde and cryoprotected in PBS containing 30% sucrose. Cryostat sections are cut at 30-μm coronally through the entire anteroposterior axis of the hippocampus and collected serially in PBS. Every 20th section through the entire hippocampus is selected in each of the animals and processed for Nissl staining. Nissl staining demonstrates the hippocampal cytoarchitecture of principal cell layers and confirmed the presence of bilateral hippocampal injury in rats after SE. The extent of neurodegeneration within different regions of the hippocampus is further assessed by NeuN immunohistochemistry and quantified from 24 to 72 h post-SE. Because the overall neurodegeneration in different regions of the hippocampus appeared mostly symmetrical between the two sides, quantification is performed on only one side. The number of cells in hippocampal subregions such as the dentate hilus (DH), CA3 and CA1 are counted and compared with controls or between various treatment groups. Reduction or lack of neuronal loss is indicative of neuroprotective potential of the test drug.

#### 7.2.2. Neurogenesis

There is strong evidence of dramatic changes in neurogenesis in the hippocampus dentate gyrus subgranular zone following SE and neuronal injury. Quantification of the extent of neurogenesis and type of cells that are born after injury would be helpful to study the pathophysiological role of neurogenesis following SE and acute neuronal injury. Interestingly, hippocampal neurogenesis is very sensitive to physiological and pathological stimuli. Certain pathological stimuli such as seizures alter both the amount and the pattern of neurogenesis [166]. Therefore, it is helpful to identify whether SE-induced changes in neurogenesis contribute to vulnerability to neurological conditions such as cognitive dysfunction, depression and epilepsy. Neurogenesis within the adult central nervous system is demonstrated using an exogenous cell tracer, 5′-bromo-2′-deoxyuridine (BrdU), in combination with endogenous neuronal markers [167–169]. Specific primary antibodies raised against these markers are widely available and their visualization is possible with the use of fluorescently tagged secondary antibodies. BrdU is a thymidine analog that incorporates into dividing cells during DNA synthesis. Once incorporated into the new DNA, BrdU will remain in place and be passed down to daughter cells following division. Typically, BrdU is injected intraperitoneally. Different survival times required by the desired experimental time-line will yield data on specific phases of neurogenesis: proliferation, differentiation and maturation. One limitation of using BrdU is uncertain penetration of the targeted cells with a uniform concentration of the compound. Thus, for experiments requiring measurements of cell proliferation, Ki67 can be used as an acceptable alternative. The protocol takes 3–5 days, allowing for sectioning and staining.

#### 7.2.3. Mossy Fiber (MF) Sprouting

Sprouting of neuronal axons including MF that contain zinc is generally visualized with Timm staining [170]. In the chronic epilepsy model, MF sprouting is indicative of epileptogenesis and is commonly identified by Timm staining of the brain section at various intervals after induction of SE. There is little or subtle change in Timm staining in acute models of SE; MF sprouting is mostly observed in chronic post-SE models of TLE.

### 7.3. Neuroinflammation Markers

Neuroinflammation is a common consequence of seizures and other neuronal injury events. Acute seizures and SE causes neuroinflammation by activating microglia, astrocytes, and induction and enhancement of inflammatory cytokines such as IL-1β, IL-6 and TNF in key brain regions such as the hippocampus [105,171–175]. Further seizure related expression of inflammatory cytokines occurs in brain regions that may undergo neuronal damage [176]. A recent report suggests that seizure-induced release of inflammatory cytokines like IL-1β from astrocytes may cause brain inflammation and damage the blood brain barrier. Neuroinflammation and its secondary consequences may partly contribute to generation of recurrence of seizures [176]. Immunohistochemistry of brain sections for specific glial markers such as glial fibrillary acidic protein (GFAP, astrocytes) and Iba-1 (microglia) is helpful to identify the pattern of neuroinflamation and relevant damage. There is a marked increase in the number of astrocytes and microglia and their processes due to inflammation. It is shown that the neurosteroid allopregnanolone can reduce inflammation in traumatic brain injury models [177,178]. Quantification of biomarkers of neuroinflammation is also very helpful to assess the course of inflammation following seizures and neuronal injury events.

## 8. Conclusions and Perspectives

There are four key areas of unmet clinical needs in epilepsy, which include (i) new AEDs for drug-resistant seizures and SE, (ii) disease-modifying drugs that prevent or ameliorate the process of epileptogenesis especially after brain injury or SE, (iii) new therapies for the comorbidities in people with epilepsy or caused by untreated SE, and (iv) new drugs for special subpopulations and age- or gender-specific epilepsies such as catamenial epilepsy (women), pediatric epilepsy (children) and geriatric epilepsy (aged population). Animals models are standard paradigms employed in drug discovery in epilepsy because seizures are a network phenomenon that cannot be created through tissue culture or simulations [179–182]. Epileptic seizures arise due to multiple factors that regulate neuronal excitability and synchrony. Most AEDs currently on the market had been advanced to clinical evaluation on the basis of their ability to block evoked seizures in one or more animal epilepsy models. SE requires rapid treatment for control of persistent seizures. Benzodiazepines are used for initial therapy, but new and effective anticonvulsants are needed for effective control of late self-sustaining or refractory SE.

A variety of models are employed for screening of new compounds for their anticonvulsant activity against SE. The ideal characteristics are:

- Rapid onset of action and intermediate duration
- Ease of administration
- Broad spectrum of activity
- Minimal sedative potential
- Aqueous solubility for i.v. solution formulations
- Effective against convulsive and non-convulsive SE
- Lack of tolerance upon repeated administration
- Possess antiseizure activity for maintenance therapy
- Should be effective when given late (>40-min) after SE onset

Pharmacological models of SE such as kainic acid, pilocarpine, and DFP are most widely used for evaluation of test agents (Table 3). Post-SE models have become the standard models to study epileptogenesis. However, there is no single animal model of epilepsy that truly predicts the clinical efficacy of new compounds. It is suggested that a battery of experimental paradigms are essential to identify the potential lead compounds. Test agents should be tested in multiple protocols including pretreatment and post-exposure paradigms. Behavioral and EEG data are analyzed for anticonvulsant efficacy as the primary outcome measure of drug effectiveness. The efficacy of drugs in terminating SE or reducing its severity is evaluated by assessing multiple parameters that include severity and duration of behavioral seizures, frequency and duration of ictal spike activity, and latency for termination of SE activity. The ability of a test agent to terminate the SE and reduce neuronal damage should be compared with a benzodiazepine such as diazepam or midazolam. The animal studies submitted for regulatory approval are generally expected to be conducted in accordance with good laboratory practice regulations.

## Figures and Tables

**Table 1:** The molecular mechanisms of current antiepileptic drugs.

Mechanism	Drug
Blockage of voltage-gated sodium channels	Phenytoin
	Fosphenytoin
	Carbamazepine
	Valproate
	Lamotrigine
	Oxcarbazepine

Enhancement of GABA inhibition	Phenobarbital
	Primidone
	Diazepam
	Lorazepam
	Clonazepam
	Tiagabine
	Valproate

Blockage of low-threshold (T-type) Ca^2+^ channels	Ethosuximide
	Gabapentin
	Valproate

Reduction of glutamate excitation	Felbamate
	Gabapentin
	Parampanel

**Table 2:** Classification of animal models of SE.

Classification	Model	References
Electrical models	Perforant pathway stimulation	[28,41–43]
	Self-sustaining stimulation	[44–46]

Chemical models	Kainic acid	[25,31,47–52]
	Pilocarpine	[26,53–58]
	Lithium-pilocarpine	[26,59,60]
	Organophosphates	[37,61–63]
	Flurothyl	[64–67]
	Cobalt-homocysteine thiolactone	[68]

Thermal models	Hyperthermia or febrile seizures	[69–74]

*In vitro* models	Low magnesium in brain slices	[75–77]
	High potassium in brain slices	[78–80]
	4-Aminopyridine in brain slices	[81–84]
	Organotypic slice cultures	[85–89]

Refractory models	Lithium-pilocarpine	[37,90,91]
	Kainic acid	[25,31,92–94]
	DFP	[37,62,95,96]

**Table 3:** General profiles of selected animal models of SE.

Feature	Kainic acid	Pilocarpine	DFP	PPS	Hyperthermia
Technical feasibility	Simple	Simple	Complex	Tedious	Tedious
Mortality rate	High	High	Medium	Low	Low
Acute neuronal injury	Severe	Severe	Severe	Moderate	Minimal
Diazepam response (early: <10-min)	Sensitive	Sensitive	Sensitive	Sensitive	Sensitive
Diazepam response (late: >40-min)	Insensitive	Insensitive	Insensitive	Sensitive	Sensitive
Neuroinflammation	Robust	Robust	Robust	Moderate	Moderate
Chronic hyperexcitability	Severe	Severe	Severe	Severe	Moderate
Neurodegeneration (>2 months post SE)	Severe	Severe	Severe	Moderate	Minimal
Spontaneous seizures (>2 months post SE)	Severe	Severe	Severe	Moderate	Minimal
Mossy fiber sprouting (>2 months post SE)	Severe	Severe	Severe	Moderate	Moderate

## Abbreviations

ACSF: Artificial cerebrospinal fluid
AD: Afterdischarge
AED: Antiepileptic drug
4-AP: 4-Aminopyridine
DH: Dentate hilus
DFP: Diisopropylfluorophosphate
EEG: Electroencephalogram
FJB: Fluoro-Jade B
HCTL: Homocysteine thiolactone
KA: Kainic acid
MF: Mossy fibers
OP: Organophosphate
PPS: Perforant path stimulation
SSL: self-sustaining limbic
SE: Status epilepticus
TLE: Temporal lobe epilepsy
